# Style Transfer–assisted Deep Learning Method for Kidney Segmentation at Multiphase MRI

**DOI:** 10.1148/ryai.230043

**Published:** 2023-09-13

**Authors:** Junyu Guo, Manu Goyal, Yin Xi, Lauren Hinojosa, Gaelle Haddad, Emin Albayrak, Ivan Pedrosa

**Affiliations:** From the Department of Radiology (J.G., M.G., Y.X., L.H., G.H., E.A., I.P.), Department of Urology (I.P.), and Advanced Imaging Research Center (I.P.), University of Texas Southwestern Medical Center, 2201 Inwood Rd, Suite 202, Dallas, TX 75390-9085.

**Keywords:** Kidney Segmentation, Generative Adversarial Network, CycleGAN, Convolutional Neural Network, Transfer Learning

## Abstract

**Purpose:**

To develop and validate a semisupervised style transfer–assisted deep learning method for automated segmentation of the kidneys using multiphase contrast-enhanced (MCE) MRI acquisitions.

**Materials and Methods:**

This retrospective, Health Insurance Portability and Accountability Act–compliant, institutional review board–approved study included 125 patients (mean age, 57.3 years; 67 male, 58 female) with renal masses. Cohort 1 consisted of 102 coronal T2-weighted MRI acquisitions and 27 MCE MRI acquisitions during the corticomedullary phase. Cohort 2 comprised 92 MCE MRI acquisitions (23 acquisitions during four phases each, including precontrast, corticomedullary, early nephrographic, and nephrographic phases). The kidneys were manually segmented on T2-weighted images. A cycle-consistent generative adversarial network (CycleGAN) was trained to generate anatomically coregistered synthetic corticomedullary style images using T2-weighted images as input. Synthetic images for precontrast, early nephrographic, and nephrographic phases were then generated using the synthetic corticomedullary images as input. Mask region–based convolutional neural networks were trained on the four synthetic phase series for kidney segmentation using T2-weighted masks. Segmentation performance was evaluated in a different cohort of 20 originally acquired MCE MRI examinations by using Dice and Jaccard scores.

**Results:**

The CycleGAN network successfully generated anatomically coregistered synthetic MCE MRI–like datasets from T2-weighted acquisitions. The proposed deep learning approach for kidney segmentation achieved high mean Dice scores in all four phases of the original MCE MRI acquisitions (0.91 for precontrast, 0.92 for corticomedullary, 0.91 for early nephrographic, and 0.93 for nephrographic).

**Conclusion:**

The proposed deep learning approach achieved high performance in kidney segmentation on different MCE MRI acquisitions.

**Keywords:** Kidney Segmentation, Generative Adversarial Network, CycleGAN, Convolutional Neural Network, Transfer Learning

*Supplemental material is available for this article*.

Published under a CC BY 4.0 license.

**Summary**

Kidney segmentation networks trained on synthetic multiphase contrast-enhanced (MCE) MRI examinations, generated from T2-weighted images using style transfer networks, achieved high performance in different dynamic phases of the original MCE MRI acquisitions.

**Key Points**

■ A cycle-consistent generative adversarial network successfully generated anatomically coregistered synthetic multiphase contrast-enhanced (MCE) MRI datasets from T2-weighted MRI acquisitions simulating the precontrast, corticomedullary, early nephrographic, and nephrographic acquisitions.
■ A deep learning segmentation model (mask region–based convolutional neural network [Mask R-CNN]) trained on a synthetic MCE MRI dataset achieved mean Dice scores between 0.91 and 0.93 and mean Jaccard scores between 0.84 and 0.86 for kidney segmentation on the original MCE MRI acquisitions.
■ Mean Dice coefficients for Mask R-CNN (0.92 ± 0.03 [SD] with postprocessing) were superior to those of direct transfer of manually annotated kidney masks from T2-weighted images to MCE MRI datasets (0.51 ± 0.15) (*P* < .001).

## Introduction

Kidney segmentation at multiparametric MRI (mp-MRI) is commonly used for evaluation of tissue characteristics (eg, enhancement, perfusion, diffusion) correlating with conditions such as hyperuricemia, acute kidney injury, and chronic kidney disease (1,2). Similarly, radiomics have been proposed to characterize renal masses at CT and MRI (3). Moreover, convolutional neural networks have been trained with T1-weighted postcontrast images, T2-weighted images, and clinical variables for predicting malignancy in renal masses (4). However, these computational efforts necessitate manual segmentation of renal masses by radiologists, which is time-consuming and rarely done in clinical practice (5). Kidney segmentation using mpMRI is challenging because of multiple acquisitions with different fields of view and orientations. Moreover, respiratory motion between different acquisitions can cause misalignment in the location of kidneys and renal masses. Misregistration is common even for similar acquisitions, such as multiphase contrast-enhanced (MCE) MRI acquisitions. Automatic segmentation of the kidney would enable training of convolutional neural networks with large cohorts.

Deep learning models trained with manual segmentation have achieved excellent performance for kidney segmentation (6). However, this approach would be prohibitively time-consuming for analysis of multiple MRI series in mpMRI examinations. Thus, automatic kidney segmentation on different MRI sequence images would provide a framework for analysis of mpMRI examinations.

Generative adversarial networks, such as the cycle-consistent generative adversarial network (CycleGAN), have been previously reported to generate synthetic medical images (7–10). For example, CycleGAN was used to generate synthetic T2-weighted brain images from T1-weighted images and vice versa. Similarly, synthetic CT images from MRI images have been proposed for several medical imaging applications (11,12). To our knowledge, deep learning methods using generative adversarial networks to generate synthetic MCE images of the abdomen or for kidney segmentation in mpMRI examinations have not been reported. The purpose of this study was to develop and validate a semisupervised style transfer–assisted automated segmentation of the kidneys in MCE MRI acquisitions.

## Materials and Methods

### MRI Datasets and Image Annotation

This retrospective, Health Insurance Portability and Accountability Act–compliant study was approved by the institutional review board. The need for informed consent was waived. MRI acquisitions from two independent cohorts of patients with known renal masses were included. Cohort 1 was previously reported in a study evaluating mpMRI for prediction of tumor histologic features (13,14). The MRI acquisition parameters at the authors’ institution are shown in Table S1. All MCE MRI examinations were performed with a three-dimensional (3D) contrast-enhanced T1-weighted spoiled gradient-echo acquisition. MCE MRI acquisitions were obtained before and after intravenous administration of gadobutrol (0.1 mmol/kg of body weight at 0.2 mL/sec) during the corticomedullary, early nephrographic, and nephrographic phases. The corticomedullary phase was timed to the late arterial phase with a power injector (15). MRI examinations performed elsewhere met minimum technical requirements (Table S2) (13,14). MRI examinations with artifacts caused by field inhomogeneity or motion were excluded from the training sessions through manual inspection.

### Cohort 1

Cohort 1 was imaged at University of Texas Southwestern Medical Center or at other institutions from January 2011 to May 2015. Overall, 102 coronal two-dimensional half-Fourier single-shot fast spin-echo T2-weighted acquisitions and 27 T1-weighted acquisitions obtained during the corticomedullary phase were included. All 102 T2-weighted datasets were annotated by a 3rd-year radiology resident (L.H.) using Philips IntelliSpace Discovery. The renal mass or cysts were not included within the kidney mask. These masks were used as ground truth for kidney segmentation training.

### Cohort 2

Cohort 2 was imaged only at University of Texas Southwestern. Twenty-three coronal MCE MRI acquisitions from July 2016 to October 2019 were included for style transfer training, each consisting of four T1-weighted acquisitions (precontrast, corticomedullary, early nephrographic, nephrographic; 92 acquisitions total).

A 2nd-year radiology resident (G.H.) manually segmented the kidneys in a sample of 20 MCE MRI examinations (80 acquisitions) using 3D Slicer (*https://www.slicer.org/*). These masks were used to evaluate model performance.

An N4 bias field correction was used for correcting field inhomogeneity (cohorts 1 and 2) (16).

### Deep Learning Method

**Figure 1:**
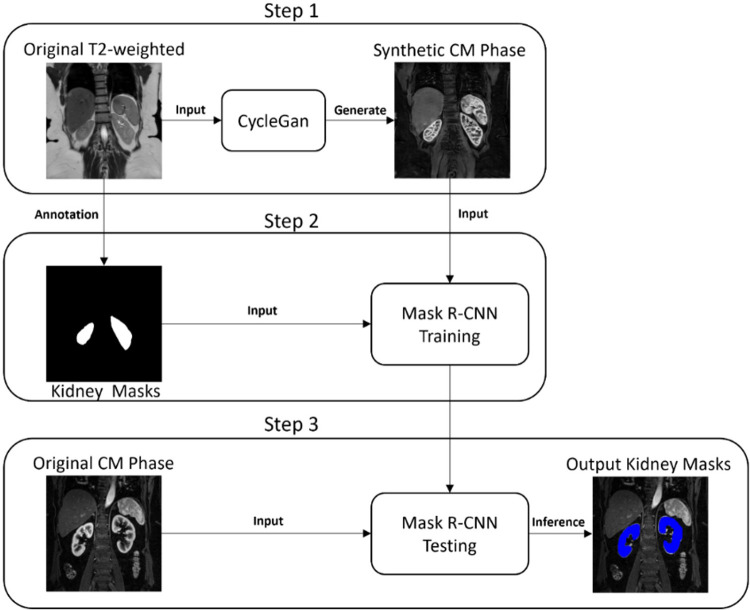
Schematic shows the deep learning method for segmentation of kidneys on T1-weighted contrast-enhanced images acquired during the corticomedullary (CM) phase. CycleGan = cycle-consistent generative adversarial network, Mask R-CNN = mask region–based convolutional neural network.

**Figure 2:**
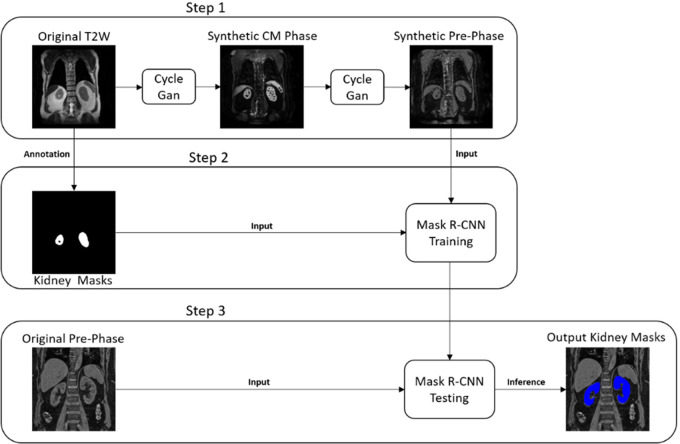
Schematic shows the deep learning method for segmentation of kidneys on T1-weighted precontrast (pre-phase) images. A similar method was used for the images acquired during the early nephrographic and nephrographic phases. CM = corticomedullary, CycleGan = cycle-consistent generative adversarial network, Mask R-CNN = mask region–based convolutional neural network, T2W = T2-weighted.

CycleGAN (17) and a mask region–based convolutional neural network (Mask R-CNN) (18) were used for semisupervised kidney segmentation in MCE MRI acquisitions (Figs 1, 2). First, anatomically coregistered synthetic images for the different MCE MRI phases were generated from the acquired T2-weighted images using CycleGAN. Second, a Mask R-CNN was trained for kidney segmentation. Finally, the Mask R-CNN performance was evaluated using an independent testing dataset (20 MCE MRI datasets from cohort 2).

***Step 1. Style transfer using CycleGAN.—*** A CycleGAN (PyTorch; *https://github.com/junyanz/CycleGAN*) model was trained to generate synthetic corticomedullary images (Cmodel 1) using the 82 T2-weighted datasets (cohort 1) as source and the 27 corticomedullary phase datasets (cohort 1) as target (Tables S3, S5). After training, anatomically coregistered synthetic corticomedullary-style images were generated using the Cmodel 1 and T2-weighted images as input (Fig 1). An attempt to use CycleGAN and T2-weighted images to generate synthetic precontrast, early nephrographic, and nephrographic images was unsuccessful because of large variations in signal intensity. Thus, we trained three different CycleGAN models (Cmodels 2, 3, and 4) to generate synthetic precontrast, early nephrographic, and nephrographic images, respectively, using the corticomedullary phase images as source (cohort 2). Details for generating style transfer images have been previously described (17).

***Step 2. Kidney segmentation using Mask R-CNN.—*** Because T2-weighted and synthetic MCE MRI images are anatomically coregistered, kidney masks created on T2-weighted images provide ground truth for training segmentation models on synthetic images. Thus, four Mask R-CNN models (Smodels 1, 2, 3, and 4) were trained using the synthetic images of each of the MCE MRI phases and the T2-weighted masks as input (Tables S4, S5).

TensorFlow was used to implement Mask R-CNN with InceptionResNetV2 *(https://github.com/tensorflow/models/tree/master/research/object_detection*). Training was performed on a high-performance computing node (Titan V100, 32 GB GPU; Nvidia). To train Mask R-CNN models, we used the gray-scale images as input and the binary kidney masks as ground truth (512 × 512). We set a batch size of four, a number of epochs of 100, an initial learning rate of 0.008 with a momentum optimizer of 0.9, and a gradient clipping by a norm of 10. The optimal model was selected based on the minimum of the validation loss. With the pretrained weights (19), the Mask R-CNN models were trained using synthetic datasets for each MCE MRI phase. Data augmentation techniques, including horizontal and vertical flips, were performed in real time during this process.

**Figure 3:**
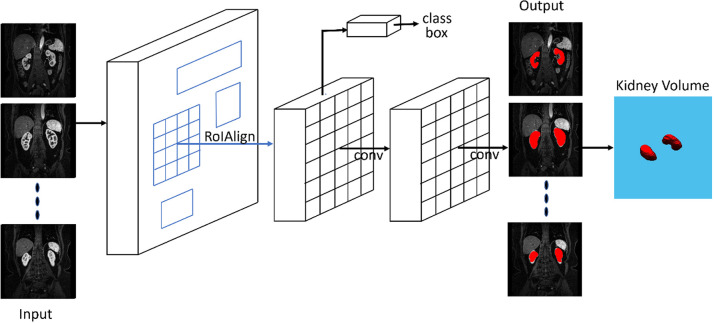
Diagram shows the mask region–based convolutional neural network (Mask R-CNN) architecture for segmentation of kidneys at multiphase contrast-enhanced MRI. T1-weighted images acquired during the corticomedullary phase are displayed. A similar method was used for precontrast images and images obtained during the early nephrographic and nephrographic phases. Conv = convolution, RoI = region of interest.

In this study, only the segmentation function in Mask R-CNN was used. A diagram of the Mask R-CNN with inputs and outputs is shown in Figure 3.

### 3D Morphologic Postprocessing

**Figure 4:**
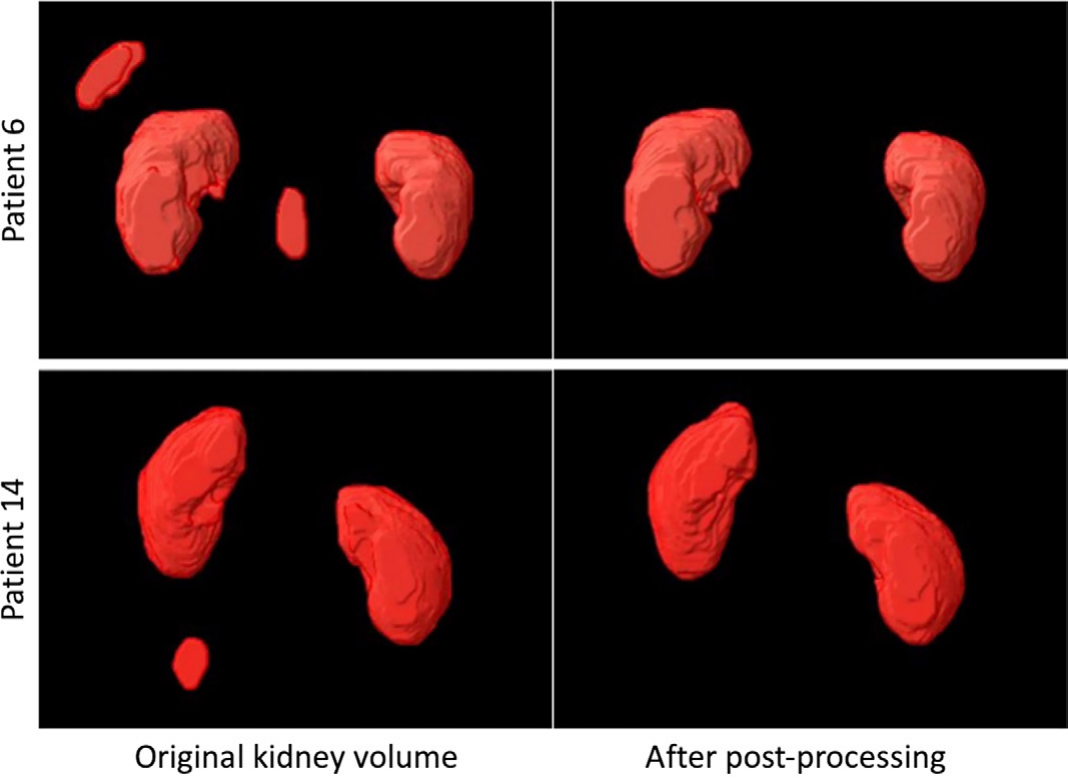
Three-dimensional morphologic postprocessing images show a comparison of the kidney mask volume before (left) and after (right) morphologic operations in two representative patients from cohort 2. In these examples, the clean, majority, and fill operations were used to remove the random voxels predicted by the algorithm.

After inference on two-dimensional MRI sections, binary kidney volumes underwent 3D morphologic postprocessing using dilation, erosion, clean, majority, and fill operations to refine segmentation to generate the final 3D kidney volume (Fig 4).

### Mask R-CNN Performance Evaluation

We evaluated the proposed semisupervised deep learning segmentation of the kidneys using an independent testing dataset (20 × 4 MCE MRI phases [precontrast, corticomedullary, nearly nephrographic, nephrographic]) from cohort 2 (Table S2). Dice and Jaccard coefficients were calculated to evaluate model performance, defined as follows (20): 
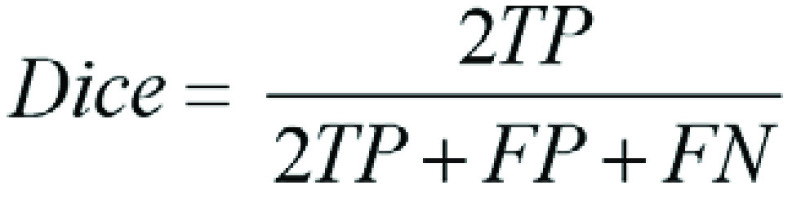


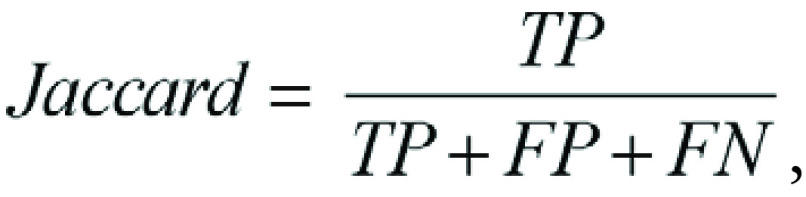
 where TP is true positive, FP is false positive, and FN is false negative.

We directly transferred masks from 20 T2-weighted examinations to 20 × 4 acquired MCE MRI phases to assess the effect of misregistration after MCE MRI images and masks were resampled to match the spatial resolution of the corresponding T2-weighted image (cohort 2). Dice and Jaccard scores were calculated between overlaid masks (direct transfer from the T2-weighted image) and original masks from four MCE MRI phases.

### Statistical Analysis

Means and SDs of Dice and Jaccard coefficients for four models were compared with and without 3D morphologic postprocessing using the Wilcoxon signed rank test.

Means and SDs of Dice and Jaccard coefficients using the direct transfer method were reported. The difference in mean Dice and Jaccard coefficients between the direct transfer method and Mask R-CNN was compared using the Wilcoxon signed rank test. To assess interreader variations of kidney masks, Dice scores were calculated for two sets of T2-weighted image masks, annotated by two different individuals with radiology training (G.H., a 2nd-year radiology resident, and E.A., a radiologist with 5 years of experience), for the 20 patients in the testing dataset. *P* < .05 was considered indicative of a statistically significant difference. All analyses were conducted using R version 4.3.0 (The R Foundation).

## Results

### Model Performance

**Figure 5:**
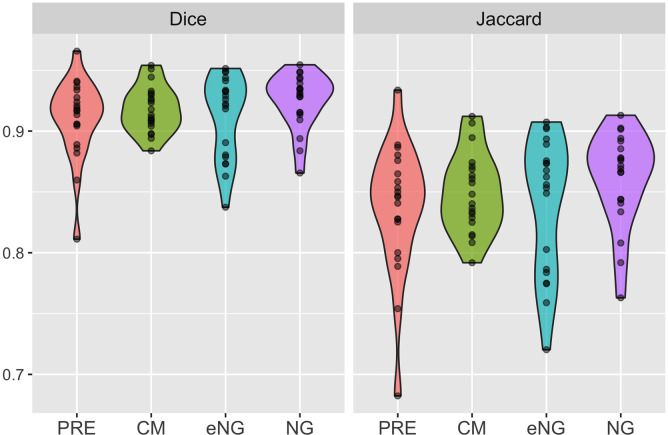
Violin plots for Dice (left) and Jaccard (right) scores show performance of the mask region–based convolutional neural network (Mask R-CNN) for segmentation of kidneys for each phase of the multiphase contrast-enhanced images in 20 patients. CM = corticomedullary, eNG = early nephrographic, NG = nephrographic, PRE = precontrast.

A total of 125 patients (mean age, 57.3 years; 67 male, 58 female) with renal masses were included in this study. Table 1 presents Dice and Jaccard scores for comparison between deep learning segmentation and manual segmentation of MCE MRI acquisitions. Mask R-CNN achieved mean Dice scores of 0.91–0.93 and mean Jaccard scores of 0.84–0.86 for the four MCE MRI acquisitions (Fig 5). The proposed method achieved a mean Dice score of 0.92 and a mean Jaccard score of 0.85 for all MCE MRI acquisitions. Figure S1 shows representative images of the patient with the lowest outlier values depicted in Figure 5. Figure S2 shows that the representative model’s predictions outperformed human manual segmentation. Figures S3 and S4 showcase representative synthetic images from different MCE phases and the corresponding acquired MCE images.

**Table 1:**
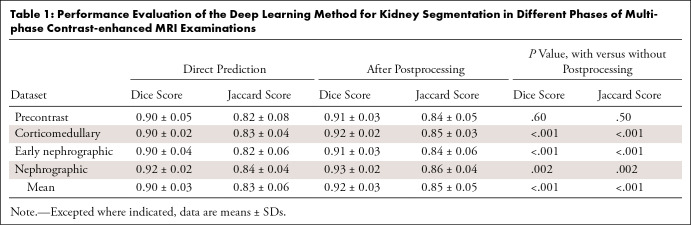
Performance Evaluation of the Deep Learning Method for Kidney Segmentation in Different Phases of Multiphase Contrast-enhanced MRI Examinations

Use of 3D postprocessing improved model Dice scores by 1.2% and Jaccard scores by 1.9% (*P* < .001) (Table 1).

**Figure 6:**
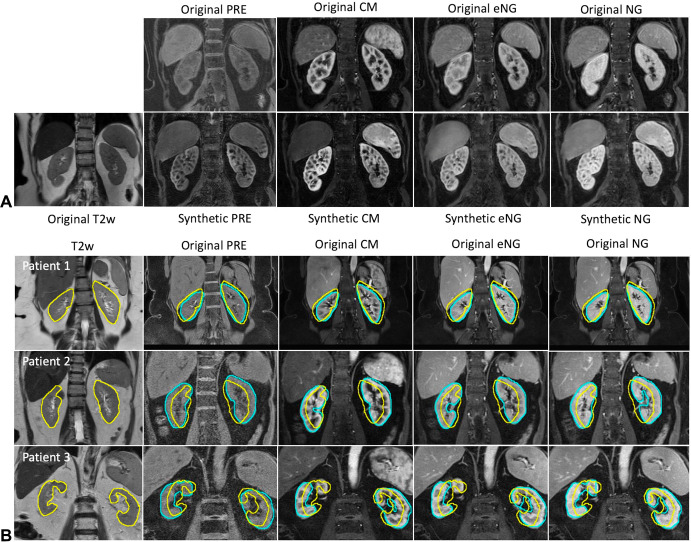
Comparison between direct transfer of kidney masks and deep learning segmentation. **(A)** Representative examples of original T2-weighted (T2w) images, original multiphase contrast-enhanced (MCE) MRI images, and synthetic MCE images generated using the cycle-consistent generative adversarial network (CycleGAN) in one patient. **(B)** Representative examples of T2-weighted and original MCE MRI acquisitions in three patients from cohort 2. The yellow regions of interest represent manual segmentation on the T2-weighted images (ie, ground truth). These regions of interest were copied and pasted onto the MCE MRI acquisitions after all MCE MRI acquisitions and masks were resampled and interpolated to match the corresponding T2-weighted acquisition in the same patient. Note the severe misregistration due to differences in anatomic location of the kidneys (eg, due to differences in respiration effort) between the different acquisitions. Cyan-colored regions of interest represent the inference masks generated by the mask region–based convolutional neural network (Mask R-CNN) in different phases of MCE MRI after training on synthetic images. Regions of interest generated by the Mask R-CNN closely outline the kidney contour despite differences in the anatomic location of the kidneys. CM = corticomedullary, eNG = early nephrographic, NG = nephrographic, PRE = precontrast.

Direct transfer of kidney masks from T2-weighted images to the original MCE MRI acquisitions yielded mean Dice and Jaccard scores of 0.51 ± 0.15 (SD) and 0.36 ± 0.13, respectively (Table 2, Fig 6), which were significantly lower than those of the Mask R-CNN (*P* < .001).

**Table 2:**
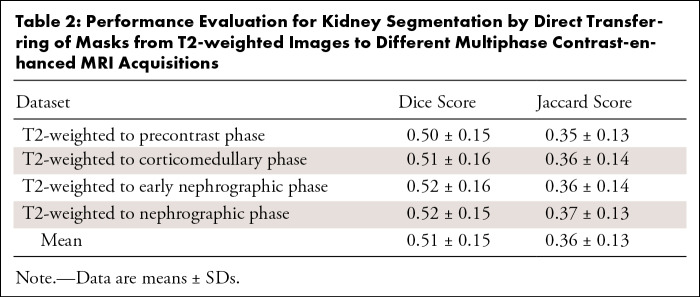
Performance Evaluation for Kidney Segmentation by Direct Transferring of Masks from T2-weighted Images to Different Multiphase Contrast-enhanced MRI Acquisitions

### Interreader Variability

When interreader variation of masks on T2-weighted images (examples in Figure S5) was evaluated, the mean Dice score between two readers was 0.90 (range, 0.86–0.93). These results were similar to the performance of the trained Mask R-CNN model (mean Dice score, 0.92).

## Discussion

This study evaluated the use of CycleGAN to automate kidney segmentation at MCE MRI. Automatic segmentation of the kidneys at mpMRI is challenged by the need to annotate multiple acquisitions with different image contrasts, orientations, and fields of view. Furthermore, respiratory misregistration between different acquisitions requires separate annotations for each acquisition. Thus, solutions that reduce the burden of this time-consuming task are needed. First, we demonstrated that CycleGAN can be used to generate synthetic MCE MRI images using T2-weighted images. Second, we confirmed that annotation on T2-weighted images can be used to train kidney segmentation models using synthetic datasets. Our results were independently validated using 80 MCE MRI datasets, with an optimal mean Dice score of 0.92 and a mean Jaccard score of 0.85. Although image registration could be another potential solution, previous studies have reported suboptimal performance for image registration algorithms (21).

Deep learning segmentation has been implemented for different organs with high Dice scores (20,22,23). Mask R-CNN is well suited for this task, although other approaches have also been successful. For example, Dice coefficients of 0.96 were achieved for segmentation of kidneys in adult polycystic kidney disease using a U-Net (20). However, this network performed kidney segmentation on T2-weighted images only. Although our approach exhibited slightly lower performance, Dice coefficients above 0.91 were observed for all four MCE MRI phases (precontrast, corticomedullary, early nephrographic, nephrographic). Of note, this was achieved without manual annotation of any MCE MRI acquisition.

Direct mask transfer from T2-weighted images to different MCE MRI phases yielded poor delineation of the kidney contour on the multiphase images, with a mean Dice coefficient of only 0.51. This is anticipated because of respiratory-induced misregistration between separate acquisitions (Fig 6). In contrast, the trained models successfully segmented the kidneys, even for patients with inconsistent respiratory breath holds.

Our study had several limitations. First, the included cohort of MRI examinations was small, and further validation on a larger dataset is necessary. Second, our method focused solely on MCE MRI examinations in the coronal plane, which is frequently used for renal mass evaluation (24). Expansion of this work to include axial acquisitions would be beneficial because these acquisitions are more commonly used for abdominal imaging. Finally, this work focused on kidney segmentation only. Future work will aim to automatically segment renal masses using the resulting regions of interest from the automatic kidney segmentation.

In conclusion, this study evaluated a semisupervised deep learning method of kidney segmentation in different MCE MRI phases. The proposed approach maintained high performance despite respiratory motion–induced misregistration. This approach can alleviate the manual segmentation burden in multiple MCE MRI acquisitions. Future work should extend the segmentation to include renal masses and kidneys in other frequently used mpMRI acquisitions.
